# First molecular and morphological characterization of *Entamoeba bovis* in domestic and wild ruminants in Italy reveals a shared lineage across host species and ecological settings

**DOI:** 10.1017/S0031182026102327

**Published:** 2026-06

**Authors:** Filippo Maria Dini, Talita Bordoni, Chiara Belli, Enrica Bellinello, Aurora Lattanzi, Roberta Galuppi

**Affiliations:** 1 Department of Veterinary Medical Sciences (DIMEVET), Alma Mater Studiorum University of Bologna, Bologna, Italy; 2 Research, Ecology and Environment Dimension (D.R.E.Am. Italia), Pistoia, Italy

**Keywords:** cattle, *Entamoeba*, goat, morphology, phylogeny, red deer, sheep

## Abstract

*Entamoeba bovis* is a common intestinal amoeba of ruminants, yet molecularly confirmed data from Europe remain limited. We investigated the occurrence, morphology and genetic variability of *E. bovis* in Italian domestic and wild ruminants and developed a rapid species-specific polymerase chain reaction (PCR) assay. Faecal samples from calves (*n* = 45), goats (*n* = 26), sheep (*n* = 11) and Italian red deer *Cervus elaphus italicus* (*n* = 25) were examined by sediment microscopy and iodine staining. Amoebic stages were detected in 57% of bovine, 61.5% of caprine, 100% of ovine and 56% of red deer samples. Across hosts, cysts were consistently uninucleate and displayed typical *E. bovis* morphology, including frequent chromatoid bodies and an iodophilic glycogen mass. Representative positives were sequenced at the 18S rRNA locus (∼800 bp), confirming *E. bovis* (98.6–99.8% identity; 100% query coverage). Maximum-likelihood phylogenetic analysis placed all Italian isolates within the *E. bovis* clade and indicated a shared lineage across domestic ruminants and red deer, with no segregation by host or ecological setting. A newly designed *E. bovis*-specific PCR (375 bp) consistently yielded a single amplicon in all sequenced positives, supporting its use for rapid screening without sequencing.

## Graphical abstract

**Figure:**
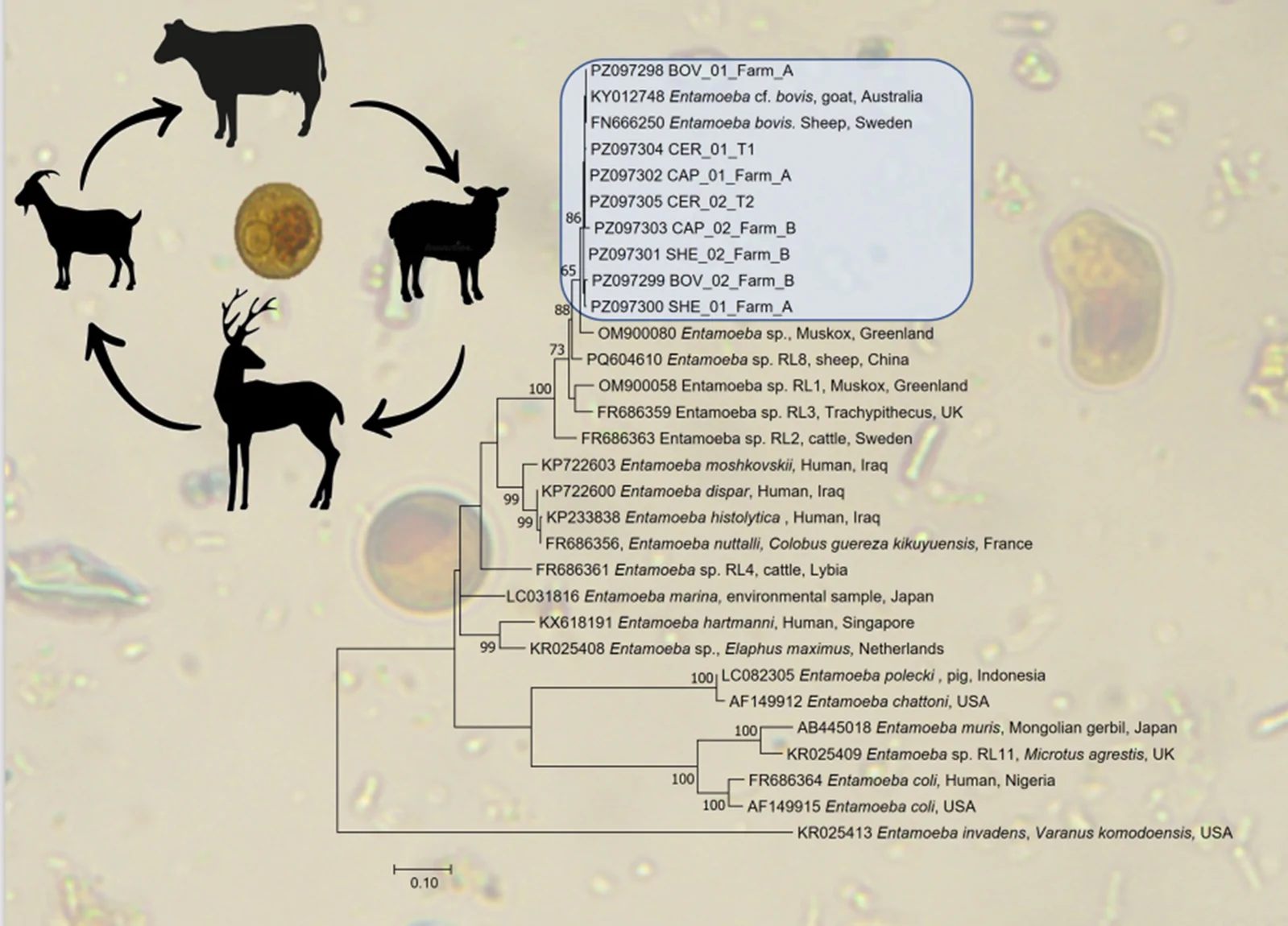

## Introduction

Different *Entamoeba* species can colonize the gastrointestinal tract of a wide range of mammalian hosts. Among these, *E. histolytica* is the only species recognized as a major health concern in humans and some other animals, whereas other *Entamoeba* species such as *E. dispar, E. moshkovoskii, E. harmanni* and *E. poleki* have been regarded as potentially pathogenic, having been occasionally associated with diarrhoea under specific conditions (Dini et al., 2025). Conversely, species infecting exclusively animal hosts have received considerably less attention, particularly with regard to their pathogenic potential. One such species is *Entamoeba bovis*.

*Entamoeba bovis* is a widely distributed amoeba, generally considered non-pathogenic, and commonly found in the intestinal tract of domestic and wild ruminants, which appear to be common hosts of uninucleated cyst-forming amoebae (Noble and Noble, 1952). Early descriptions of *E. bovis* were based exclusively on microscopic observations, with records dating back to the early 20th century. Traditional morphological studies such as that of Kingston and Stabler (1978) on *Odocoileus virginianus* (white-tailed deer), which reported small trophozoites with a single endosome and uninucleate cysts containing bar-shaped chromatoid bodies. However, early taxonomy was hampered by reliance on a limited set of morphological characters (such as cyst nuclear number, chromatoid body morphology and host association) leading to substantial confusion and synonymy among morphologically similar species. During the first quarter of the 20th century, numerous species names were assigned to amoebae of the genus *Entamoeba*, often primarily on the basis of their occurrence in new hosts (Dobell, 1919).

Accordingly, in goats, several *Entamoeba* species have been historically reported, including *E. debliecki* (syn. *E. polecki*) from the large intestine, *E. caprae* from the stomach and intestine, *E. dilimani* and *E. wenyoni* from the intestine. In sheep, both *E. ovis* and *E. capreae* have been described. Notably, the morphological characteristics of these amoebae are highly similar, with the exception of *E. wenyoni*, which was described as having small (<10 µm), octonucleated cysts. Moreover, for *E. caprae*, cyst stages have not been described, and only trophozoites have been reported (Hoare, 1959).

Recent integrative studies combining microscopy with molecular approaches have substantially reshaped the understanding of *Entamoeba* diversity in ruminant hosts, whether domestic or wild, highlighting the central role of molecular confirmation for reliable species-level identification, particularly through 18S rRNA gene sequencing (Stensvold et al., 2010).

Currently, genetic data available in public databases indicate that *Entamoeba* sequences obtained from domestic and wild ruminants are dominated by *E. bovis*, together with several distinct ribosomal lineages closely related to this species, designated RL1–3 and RL8. These lineages are phylogenetically allied to *E. bovis* and are characterized by the availability of more than 80% of the SSU rDNA gene sequence, differing from previously described sequences by 5% or more, while lacking corresponding morphological characterization (Jacob et al., 2016).

Collectively, these findings suggest that much of the historical species diversity of *Entamoeba* reported in ruminant hosts on the basis of microscopy alone (often distinguished primarily by host species and gastrointestinal niche) may in fact reflect genetic variation within a limited number of closely related taxa, with *E. bovis* representing the predominant lineage currently supported by molecular evidence.

Despite several studies conducted in Asia, Australia and North America, data on the occurrence and diversity of *Entamoeba* spp. in European ruminants remain scarce. To date, no morphological or molecular characterization of *Entamoeba* species colonizing ruminants has been reported from Italy.

Here, we provide the first evidence of *Entamoeba* presence in Italian ruminants. Our study integrates detailed morphological observations with molecular characterization based on 18S rRNA gene sequencing to describe parasite morphology and assess genetic variability across multiple domestic and wild ruminant hosts.

## Materials and methods

### Sample collection, study areas and coprological examination

Faecal samples from cattle, sheep, goats and an Italian subspecies of red deer were collected in the context of routine coprological examinations performed at the Parasitology Laboratory, Department of Veterinary Medical Sciences, University of Bologna (Italy).

For domestic ruminants, samples were obtained through opportunistic sampling from multiple farms for which the Parasitology Laboratory provides routine parasitological diagnostic services, either as part of scheduled coprological screening programmes or following clinical suspicion of parasitic infections.

Forty-five bovine samples were collected in 2024 from 2 farms located in northern Italy, within the framework of routine coprological screening of calves. The 2 farms represented distinct production systems and environmental settings, including 1 dairy farm located in an anthropized lowland area (Farm A) and 1 beef farm situated in a more isolated hilly environment (Farm B). The age of calves ranged from 14 to 176 days.

Caprine samples originated from 2 distinct holdings in north-central Italy. One was a small-scale farm (Farm A) located in the urbanized area of the Province of Bologna (Emilia-Romagna), from which 5 pooled samples were analysed (3 in 2024 and 2 in 2025), each representing a different age/sex category (kids, juveniles, adult females and adult males). The second was a farm (Farm B) located in a mountainous rural area of the Province of Florence (Tuscany), from which faecal samples from 21 individual goats were examined.

Ovine samples were obtained in 2024 from 2 farms in the Province of Bologna: 1 located in an urbanized area near the city (Farm A), from which 6 individual samples were collected, and a second located in a more rural hilly area (Farm B), from which 5 individual samples were analysed.

In contrast, faecal samples from wild ruminants (red deer) were collected during 2 targeted capture campaigns conducted within a protected natural reserve in the Emilia-Romagna region (T1 and T2): The Bosco della Mesola, on the southern edge of the Po Delta, in the province of Ferrara, is a wooded area of 1058 hectares characterized by flora typical of sandy areas, Mediterranean scrub and wetlands. Within the area, traffic is limited to dirt roads that are accessible by off-road vehicles. In October 2024, it was possible to perform, during 2 separate events, 2 opportunistic samplings of faeces from Italian red deer during the free-ranging capture operations to relocate a pool of animals to the Serre Regional Nature Park, Calabria. A total of 25 individuals were sampled, including 10 during the first sampling event and 15 during the second sampling period. Deer capture and handling was authorized by Raggruppamento Carabinieri Biodiversità, management body of the Natural State Reserve, under permit number 84/13-4/2022 signed on 17 January 2023, following the procedures reported in the feasibility study by ISPRA (National Institute for Environmental Protection and Research) on 9 December 2021. During chemical immobilization, the faeces used for this study were collected directly from the rectal ampulla with a glove. They were then placed in plastic bags and kept at refrigerated temperature until analysis at the University of Bologna.

At the time of collection, faecal consistency varied among the species studied. Calf samples were generally soft to semi-formed, consistent with a milk-based diet, whereas samples from caprine, ovine and red deer species appeared as typical well-formed, solid pellets.

All samples were processed by double sedimentation concentration, and an aliquot of the resulting sediment was used to prepare 2 direct smears: 1 with saline, and the other stained with Dobell’s iodine.

## *Molecular confirmation*, E. bovis *primers design and phylogeny*

One representative positive sample from each farm or sampling event was selected for DNA extraction using the NucleoSpin® Stool kit (Macherey-Nagel), according to the manufacturer’s instructions.

DNA extracts were subjected to endpoint polymerase chain reaction (PCR) amplification targeting the 18S rRNA gene using the primer pair Enta-F (5′- ATG CAC GAG AGC GAA AGC AT-3′) (Hamzah et al., 2006) and E1-R (5′-GTACAAAGGGCAGGGACGTA-3′) (Khairnar et al., 2007), which amplifies an approximately 800 bp fragment of *Entamoeba* spp. 18S rDNA. Each reaction was performed in a final volume containing 2.5 μL of template DNA and 0.25 μM of each primer. PCR cycling conditions consisted of an initial denaturation step at 96 °C for 2 min, followed by 30 cycles of denaturation at 92 °C for 60 s, annealing at 56 °C for 60 s and extension at 72 °C for 90 s, with a final extension step at 72 °C for 7 min.

To facilitate the molecular identification of *E. bovis* complex species, species-specific primers were designed targeting the18S rRNA. Reference nucleotide sequences of *E. bovis* (OM900107) and closely related *Entamoeba* species (*E. histolytica* (AB426549), *E. dispar* (KP722600), *E. coli* (FR686364), *E. nuttalli* (FR686356) and *E. invadens* (KR025413)) were retrieved from the NCBI GenBank database. To minimize the risk of non-specific amplification, host genomes (*Bos taurus, Capra hircus, Ovis aries* and *Cervus elaphus*) were included during in silico specificity assessment.

Multiple sequence alignment was performed using Clustal Omega in order to identify regions conserved within *E. bovis* but divergent from other *Entamoeba* species. A suitable target region was selected for primer development using the Primer Design tool implemented in GenBank.

Primer design parameters included a length of 18–25 bp, GC content between 40% and 60%, and melting temperatures (Tm) ranging from 55 °C to 65 °C. The resulting primer pair was: Enta_bovis-F (5′-ACAGAAGTCCGGTAACGC-3′) and Enta_bovis-R (5′-CATCCATCCATACGGACA-3′), yielding an expected amplicon size of 375 bp. The forward and reverse primers exhibited GC contents of 55.6% and 50%, respectively, with calculated melting temperatures of 57.02 °C and 53.34 °C.

PCR reactions were carried out in a final volume of 25 µL containing 2.5 µL of template DNA, 0.15 µL of each primer (50 pmol), 12.5 μL 2× Dream Taq Hot Start Green PCR Master Mix (Thermo Scientific) and 9.5 μL of sterile nuclease free water. Thermal cycling consisted of an initial denaturation at 96 °C, followed by 30 cycles of denaturation at 94 °C for 3 min, annealing at 56 °C for 90 s and extension at 72 °C for 2 min, with a final extension step at 72 °C for 7 min. As a positive control, a previously sequenced *E. bovis*–positive sample obtained from a goat was included in each PCR run.

All amplification products were resolved by electrophoresis on 1.5% agarose gels stained with SYBR™ Safe (Invitrogen). For sequencing, amplicons were excised from the gel, purified using the NucleoSpin® Gel and PCR Clean-up kit (Macherey-Nagel, Düren, Germany), and sequenced on an ABI 3730 DNA Analyzer (StarSEQ, Mainz, Germany). Chromatogram trace files were assembled using ContigExpress (Vector NTI Advance 11, Invitrogen, Carlsbad, CA, USA), and consensus sequences were compared with reference sequences available in GenBank using BLAST (accessed on 30 October 2025).

Phylogenetic analysis was conducted to infer the evolutionary relationships among *Entamoeba* isolates obtained from different hosts and geographic locations. Multiple sequence alignment was performed using BioEdit version 7.2.5 (Hall, 1999). The alignment included 18S rRNA gene sequences generated in the present study together with reference sequences of *Entamoeba* spp. retrieved from the GenBank database.

The best-fitting nucleotide substitution model was determined using MEGA version 7 (Kumar et al., 2016) based on the lowest Bayesian Information Criterion score. Phylogenetic relationships were inferred using the maximum-likelihood (ML) method under the Tamura 3-parameter model with gamma-distributed rate variation and a proportion of invariant sites (TN92 + G + I). Branch support was assessed by bootstrap analysis with 1000 replicates.

The resulting phylogenetic tree was used to assess the genetic relationships between isolates obtained in this study and previously characterized *Entamoeba* lineages.

## Results

### Occurrence of Entamoeba sp. in ruminants

Overall, 26 out of 45 (57%) bovine faecal samples examined by sediment microscopy showed the presence of cysts and/or trophozoites morphologically consistent with *Entamoeba* spp. Both investigated farms tested positive for *Entamoeba*. Specifically, 22 out of 37 calves tested positive from the dairy farm in the urbanized area (Farm A), and 4 out of 8 calves tested positive from the beef farm in the hilly area (Farm B). Amoebic stages were detected exclusively in animals older than 30 days, whereas no cysts or trophozoites were observed in younger calves.

Among goats, 16 out of 26 examined samples (61.5%) were microscopically positive for amoebic stages. In the mountainous farm, 12 of 21 animals tested positive, while in the lowland farm located near the urban area, 4 out of 5 samples were positive. In the latter holding, positive samples were recorded across all age and sex categories, including kids, juveniles, adult females and adult males.

All ovine samples examined (11/11, 100%), collected from 2 farms located in different environmental settings (1 near the city and 1 in an isolated hilly area), were positive for *Entamoeba* cysts and/or trophozoites.

In red deer, *Entamoeba* stages were detected in 14 of 25 examined individuals (56.0%) by faecal microscopy, including 5 of 10 animals sampled during the first sampling period and 9 of 15 sampled during the second sampling period.

### Morphological characterization

**Figure 1.**
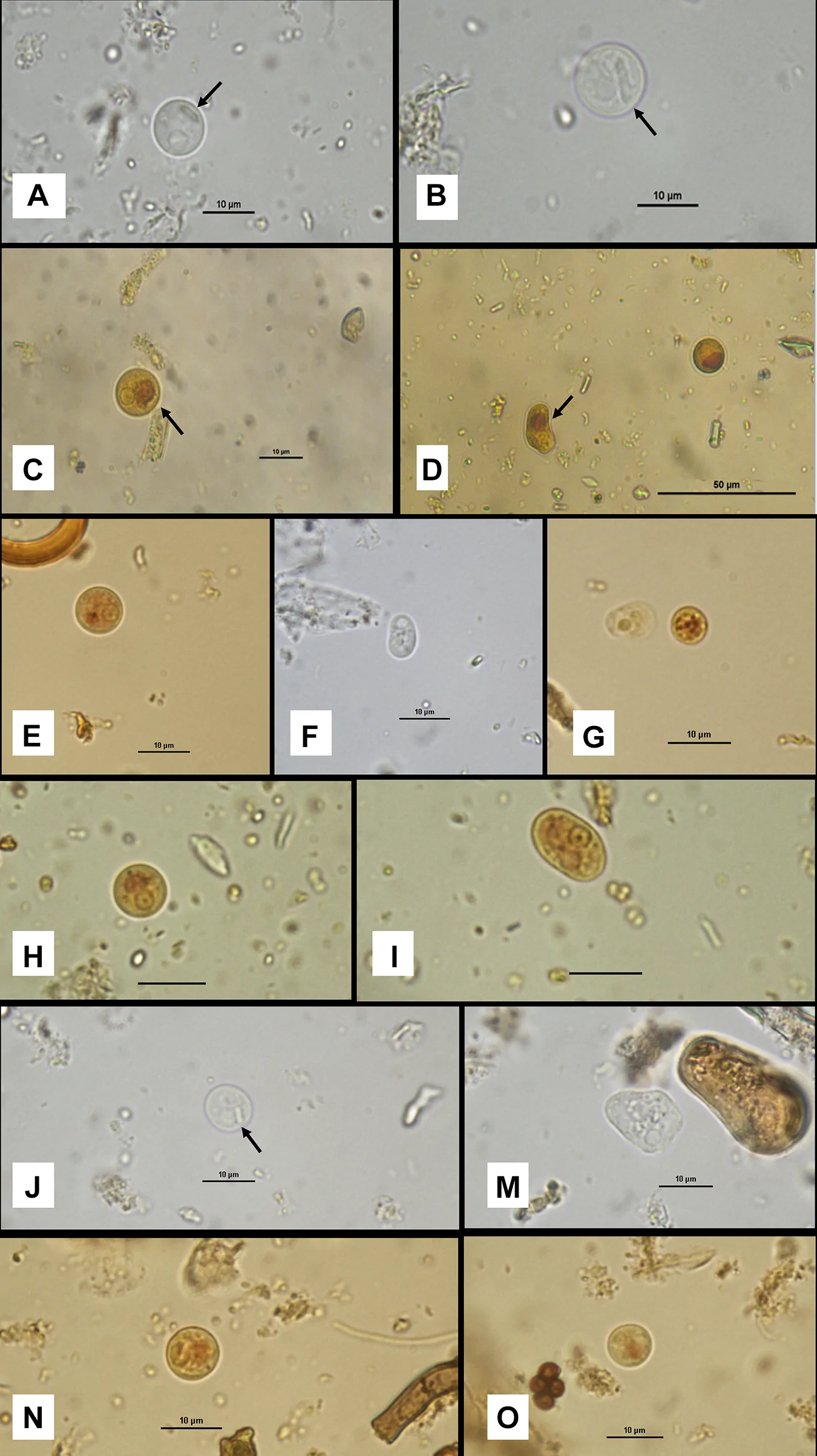
Morphological features of cysts and trophozoites of *Entamoeba* sp. From different host species: cattle (A–D), goat (E–G), sheep (H–I) and red deer (L–O). (A, B) Cysts from cattle observed in saline solution, showing chromatoid bodies (arrows). 1000 × magnification; scale bar: 10 µm. (C) Cyst from a bovine sample with clearly visible nuclear morphology and a well-defined iodophilic glycogen mass (arrow). Iodine staining. 1000 ×; scale bar: 10 µm. (D) Trophozoite (left) and cyst (right) from cattle; the arrow indicates a prominent glycogen mass in the trophozoite. Iodine staining. 400 ×; scale bar: 50 µm. (E) Cyst from goat showing typical nuclear morphology and a small glycogen mass. Iodine staining. 1000 ×; scale bar: 10 µm. (F) Trophozoite from goat with piriform shape and granular cytoplasm. Saline solution. 1000 ×; scale bar: 10 µm. (G) Trophozoite (left) and cyst (right) from goat, highlighting the markedly granular appearance of the cyst cytoplasm. Iodine staining. 1000 ×; scale bar: 10 µm. (H) Cyst from sheep faecal sample with evident nuclear morphology and a poorly defined glycogen mass. Iodine staining. 1000 ×; scale bar: 10 µm. (I) Trophozoite from sheep with clearly visible nucleus. Iodine staining. 1000 ×; scale bar: 10 µm. (L) Cyst from red deer with a clearly visible chromatoid body (arrow). Saline solution. 1000 ×; scale bar: 10 µm. (M) Trophozoite from red deer showing irregular shape, finely granular cytoplasm and visible nucleus. Saline solution. 1000 ×; scale bar: 10 µm.

In cattle, cysts exhibited a mean diameter of 8.0 µm (range: 5.7–10.9 µm) and were consistently uninucleate. The nucleus measured on average 2.6 µm in diameter (range: 1.8–3.0 µm) and displayed a rounded morphology with a centrally located karyosome and uniformly distributed peripheral chromatin. Elongated chromatoid bodies with rounded ends were frequently observed (Figure 1A, B, L; arrowed). A well-defined iodophilic glycogen mass was present within the cyst cytoplasm, with a mean diameter of 3.45 µm (range: 2.2–4.6 µm) (Figure 1C; arrowed).

Trophozoites from bovine samples showed a mean length of 9.5 µm (range: 6.5–12.8 µm) and a mean width of 6.6 µm (range: 5.8–7.2 µm). Nuclear morphology was consistent between cystic and trophic stages, exhibiting a round nucleus with a central karyosome and evenly distributed peripheral chromatin. An iodophilic glycogen mass was also observed in trophozoites (Figure 1D; arrowed).

In goats, cysts had a mean diameter of 7.5 µm (range: 5.2–10.4 µm) (Figure 1E). Trophozoites measured on average 7.9 µm (range: 5.0–10.2 µm) in length and 5.8 µm (range: 5.3–6.7 µm) in width and typically exhibited a pyriform shape, only rarely showing membrane protrusions (Figure 1F). The nucleus had a mean diameter of 2.2 µm (range: 1.8–2.8 µm) and was characterized by a central karyosome with regularly distributed peripheral chromatin. Some cysts displayed cytoplasm rich in fine granular vacuoles (Figure 1G, right). Chromatoid bodies were visible. The iodophilic glycogen mass, well defined in cysts but less evident in trophozoites, had a mean diameter of 3.1 µm (range: 1.9–4.0 µm).

In sheep, uninucleate cysts exhibited a mean diameter of 8.5 µm (range: 5.7–12.0 µm) (Figure 1H). The nucleus measured on average 2.4 µm in diameter (range not recorded) and showed a central karyosome with uniformly distributed peripheral chromatin. The iodophilic glycogen mass had a mean diameter of 3.07 µm (range: 1.9–3.3 µm). Chromatoid bodies were visible. Trophozoites were larger, with a mean length of 10.4 µm (range: 6.0–12.4 µm) and a mean width of 8.6 µm (range: 6.2–10.0 µm) (Figure 1I). In trophozoites, the iodophilic mass was often less organized and more difficult to measure accurately.

In red deer samples, cysts had a mean diameter of 7.8 µm (range: 5.2–10.3 µm), with a nucleus measuring on average 2.9 µm in diameter (range: 1.9–3.3 µm) (Figure 1L, N, O). The nucleus exhibited the same morphological features observed in the other hosts, namely a rounded shape with a centrally located karyosome and uniformly distributed peripheral chromatin. An iodophilic vacuole with a mean diameter of 4.3 µm (range: 2.5–4.9 µm) was present within the cyst cytoplasm. Chromatoid bodies were visible (Figure 1L; arrowed). Trophozoites exhibited a more elongated morphology, with a mean length of 9.4 µm (range: 6.2–12.0 µm) and a mean width of 6.6 µm (range: 5.6–7.3 µm) (Figure 1M). In trophozoites, the iodophilic vacuole appeared less organized.

### Molecular results

All selected samples subjected to PCR amplification targeting the 18S rDNA gene using the primer pair Enta-F/E1-R yielded positive results, producing a single amplicon of approximately 800 bp, consistent with the expected fragment size for *Entamoeba* spp. No amplification was observed in the negative control included in each PCR run.

The analysed samples represented isolates obtained from different host species (cattle, goats, sheep and red deer), distinct farm management systems (urbanized and rural environments), and, in the case of red deer, different sampling periods.

Direct sequencing of the PCR products generated high-quality chromatograms for all amplicons, with well-resolved peaks and no evidence of ambiguous base calls after manual inspection and trimming. BLAST analysis of the resulting consensus sequences against the NCBI GenBank database confirmed their identity as *E. bovis*, with sequence identity values ranging from 98.6% to 99.8% depending on the reference sequence, and query coverage of 100%. The closest matches corresponded exclusively to previously characterized *E. bovis* isolates. Detailed information on sequence identity, and corresponding GenBank accession numbers for both reference sequences and sequences generated in this study is provided in Table 1.

**Table 1.** Molecular identification of *Entamoeba bovis* isolates based on 18S rRNA gene sequencing. Host species, sample origin, amplicon size and BLAST results are shown for all successfully sequenced samples Table 1 long description.

Sample ID	Host	Location	Best BLAST match	Identity (%)	Reference accession	This study accession
BOV_01	Cattle	Farm A, urban	*E. bovis*	99.4	PQ6044611	PZ097298
BOV_02	Cattle	Farm B, rural	*E. bovis*	99.0	PQ6044615	PZ097299
SHE_01	Sheep	Farm A, urban	*E. bovis*	99.3	KY012748	PZ097300
SHE_02	Sheep	Farm B, rural	*E. bovis*	98.6	FN666248	PZ097301
CAP_01	Goat	Farm A, urban	*E. bovis*	99.8	OM900062	PZ097302
CAP_02	Goat	Farm B, rural	*E. bovis*	98.9	OM900085	PZ097303
CER_01	Red Deer	Reserve T1	*E. bovis*	99.8	OM415394	PZ097304
CER_02	Red Deer	Reserve T2	*E. bovis*	99.8	OM415413	PZ097305

To further confirm species-level identification, all DNA samples were also subjected to endpoint PCR using the newly designed *E. bovis*-specific primers Enta_bovis-F and Enta_bovis-R, targeting a 375 bp fragment of the 18S rDNA gene. All samples yielded a single, well-defined amplicon of the expected size (∼375 bp), with no evidence of nonspecific amplification products or secondary bands. Negative controls remained consistently negative, confirming the absence of contamination.

Sequencing of these species-specific amplicons also produced high-quality chromatograms, and subsequent BLAST analysis confirmed their identity as *E. bovis*, showing sequence identity values approaching or reaching 100% and complete query coverage with multiple *E. bovis* reference sequences available in GenBank (e.g. OM15364). The slightly higher identity values observed for this shorter fragment compared to the longer amplicon likely reflect the reduced probability of detecting point mutations across a shorter sequence length, whereas the longer fragment allowed the identification of occasional single-nucleotide substitutions among isolates. A representative sequence obtained with the *E. bovis* species-specific assay has been submitted to Genbank with the accession number PZ097306.

Given the presence of minor nucleotide polymorphisms detected within the longer 18S rDNA gene fragment, phylogenetic analysis was conducted using these sequences to investigate the evolutionary relationships among isolates obtained from different host species and ecological settings.

**Figure 2.**
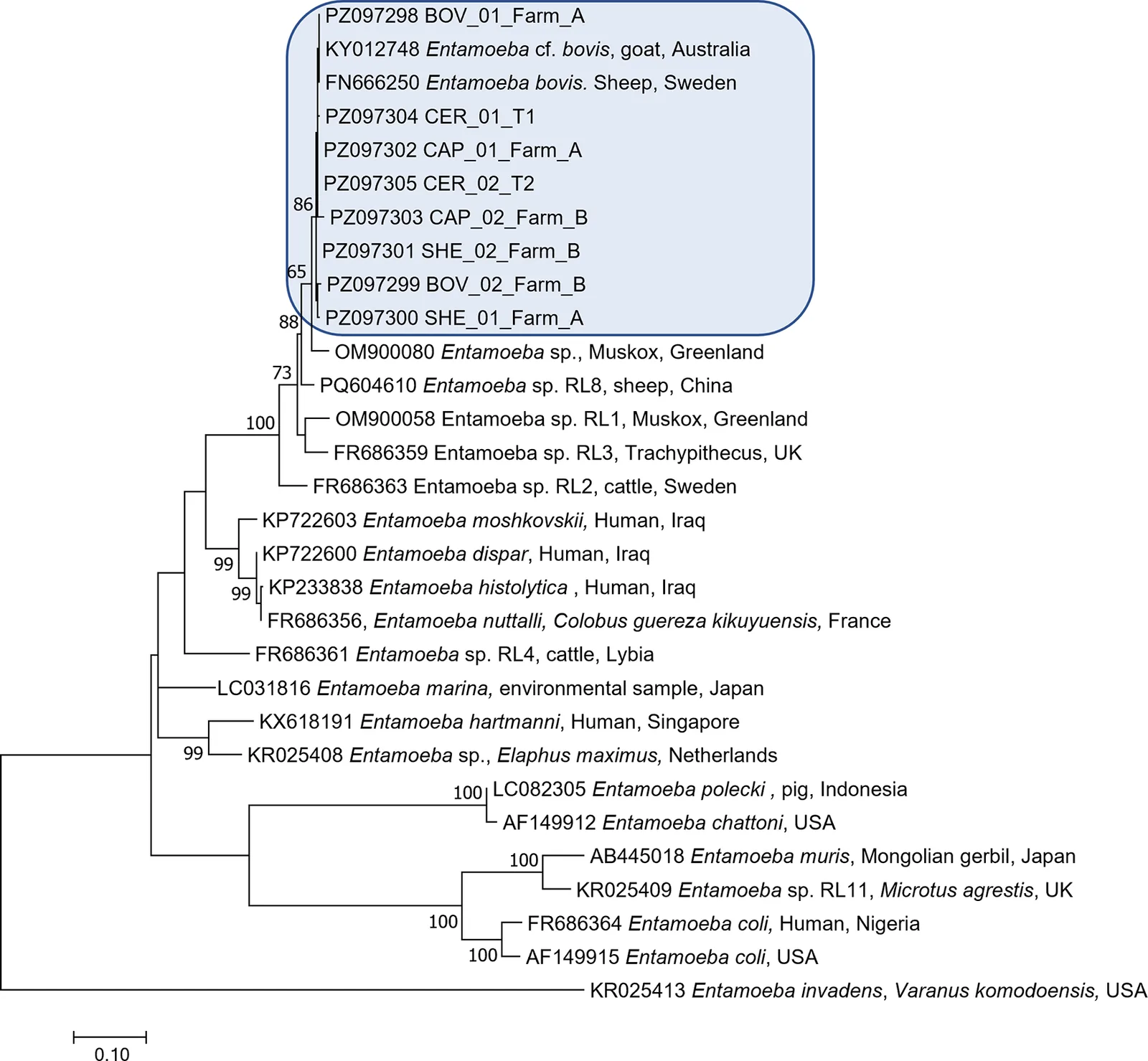
Maximum-likelihood phylogenetic tree inferred from partial 18S rRNA gene sequences of *Entamoeba* spp., Constructed using the Tamura 3-parameter model with gamma distribution and invariant sites (TN92 + G + I) in MEGA 7. Bootstrap values (>50%) based on 1000 replicates are shown at the nodes. Sequences obtained in this study (highlighted in blue) clustered within the *Entamoeba bovis* lineage alongside reference sequences from GenBank. Figure 2 long description.

Phylogenetic analysis based on partial 18S rRNA gene sequences placed all isolates obtained in the present study within the *E. bovis* clade, in close association with reference sequences annotated as *E. bovis*, including KY012748.1 (*Entamoeba* cf. *bovis* from cattle) and FN666250.1 (*E. bovis* isolate from sheep). All study-derived sequences, including BOV_01_Farm_A, BOV_02_Farm_B, SHE_01_Farm_A, SHE_02_Farm_B, CAP_01_Farm_A, CAP_02_Farm_B, CER_01_T1 and CER_02_T2, clustered within this lineage, confirming their assignment to *E. bovis* (Figure 2).

The *E. bovis* cluster was clearly separated from other closely related but distinct *Entamoeba* sequences included in the analysis. In particular, the sequence OM900080.1 (*Entamoeba* sp. from Muskox, Greenland) formed a separate branch outside the *E. bovis* cluster, with the node separating this sequence from the *E. bovis* lineage supported by a bootstrap value of 65%. Similarly, additional *Entamoeba* sp. sequences formed distinct phylogenetic groups, supported by bootstrap values of up to 88%, further confirming the genetic distinction between *E. bovis* and other related but unassigned ribosomal lineages.

Within the *E. bovis* clade, the sequences generated in this study formed a coherent phylogenetic group together with reference *E. bovis* sequences. An internal node supported by a bootstrap value of 86% separated a subset of isolates, including CAP_02_Farm_B, SHE_02_Farm_B, BOV_02_Farm_B and SHE_01_Farm_A, from the remaining isolates and reference sequences. However, this internal structuring did not correspond to host species, farm origin or ecological setting, as isolates from different hosts (cattle, sheep, goats and red deer) and environments (urban and rural farms), were interspersed within the same phylogenetic lineage.

Overall, the short branch lengths and clustering pattern observed indicate low genetic divergence among *E. bovis* isolates, consistent with intraspecific sequence variability within a single phylogenetic ribosomal lineage.

## Discussion

In the present study, cysts and trophozoites morphologically consistent with *Entamoeba* spp. were detected in multiple domestic and wild ruminant species and were molecularly confirmed as *E. bovis* based on 18S rRNA gene sequencing. The observed morphological features were consistent with classical descriptions of this species, with trophozoites appearing small and containing a single vesicular nucleus with a centrally located karyosome and evenly distributed peripheral chromatin. Cysts were uninucleate and frequently displayed characteristic elongated chromatoid bodies, often accompanied by a prominent iodophilic glycogen mass, in agreement with early morphological descriptions (Noble and Noble, 1952; Hoare, 1959; Kingston and Stabler, 1978).

Historically, several *Entamoeba* species have been described in ruminants based primarily on host specificity and anatomical site of colonization, often in the absence of molecular confirmation. This reliance on morphological criteria alone has contributed to taxonomic uncertainty and possible overestimation of species diversity. More recent molecular studies have clarified that *E. bovis* represents the most common *Entamoeba* species colonizing ruminants, while additional ribosomal lineages of uncertain taxonomic status have also been reported (Jacob et al., 2016).

These findings highlight the importance of integrating morphological observations with molecular characterization for accurate species identification. This approach is particularly critical for amoebae, whose cyst stages may exhibit substantial morphological similarity across species. In ruminants, uninucleate cysts of *E. bovis*, especially when containing a prominent iodophilic glycogen mass, may resemble immature stages of *Entamoeba histolytica*-like species or, less specifically, cysts of other intestinal amoebae such as *Iodamoeba bütschlii*, potentially leading to misidentification in the absence of molecular confirmation (Fotedar et al., 2007; Dini et al., 2025).

Regarding its clinical significance, *E. bovis* is traditionally considered a commensal, as robust studies investigating its pathogenicity in ruminants are currently lacking. During sampling, no overt clinical signs were observed in positive animals. The soft to semi-formed faecal consistency in calves was attributed to their liquid-based diet rather than infection. Furthermore, in all other species, *E. bovis* was detected in subjects with typical, well-formed pellets, suggesting that this species does not significantly alter intestinal function under normal conditions.

The occurrence of *E. bovis* observed in the present study is consistent with previous reports indicating that this species is widely distributed among domestic and wild ruminants worldwide. Molecular surveys conducted in Asia have demonstrated a high prevalence of *E. bovis* across multiple farm animal species, with particularly high detection rates reported in livestock from the Qinghai Tibetan Plateau, where *E. bovis* represented the predominant *Entamoeba* species identified (Ai et al., 2021; Ren et al., 2021). Similarly, *E. bovis* has been confirmed as the main *Entamoeba* species infecting alpacas in China (Gao et al., 2021) and cattle in Japan, where molecular characterization of microscopically detected cysts confirmed their identity as *E. bovis* (Matsubayashi et al., 2018).

Beyond domestic livestock, this species has also been reported in wild ruminants from diverse geographic regions, including muskoxen and sheep in Greenland (Stensvold et al., 2023) and wild deer in Australia, where *E. bovis* and closely related ribosomal lineages were detected in both wild and domestic hosts (Huaman et al., 2022), and in China (Liu et al., 2022).

In addition to domestic ruminants in the present study, *E. bovis* was detected in the Italian red deer (*Cervus elaphus italicus*), an endemic subspecies characterized by a distinctive ecological and conservation history. This population represents the only native red deer lineage remaining on the Italian peninsula and has persisted in long-term geographic isolation within Bosco della Mesola State Nature Reserve, a protected coastal habitat in the Po Delta Regional Park (Zachos et al., 2014). Following a severe population decline during the 20th century, targeted conservation and management programmes have enabled its recovery; however, the population remains geographically restricted and ecologically distinct.

Within the same reserve, fallow deer (*Dama dama*) have been present since the Renaissance period. Their population has undergone marked demographic expansions followed by repeated control interventions throughout the 20th and 21st centuries, due to their ecological impact and competitive interactions with the Italian red deer (Mattioli et al., 2003; Lovari and Nobili, 2010; Raganella Pelliccioni et al., 2013).

As part of ongoing conservation and reintroduction programmes, health monitoring activities have been conducted during capture operations. In this context, faecal examination revealed the presence of amoebic stages morphologically indistinguishable from those observed in domestic ruminants. Molecular analysis further confirmed these isolates as *E. bovis*. The phylogenetic analysis revealed that *E. bovis* isolates obtained from Italian red deer clustered within the same lineage as those detected in domestic ruminants, including cattle, sheep and goats, with no evidence of host-associated segregation. Notably, red deer isolates grouped together with sequences obtained from livestock raised under both urbanized intensive farming systems and more isolated hilly and mountainous environments.

This finding suggests that a shared *E. bovis* lineage circulates among wild and domestic ruminants across different ecological contexts. The presence of closely related sequences in red deer inhabiting a protected natural reserve and in domestic animals raised in environments with varying degrees of wildlife interface indicates that this amoeba is not restricted to a specific host or habitat type.

Similar findings have been reported in other wild ruminant populations, supporting the ecological flexibility and broad host range of *E. bovis*. In Greenland, Stensvold et al. (2023) detected *E. bovis* and closely related ribosomal lineages in muskoxen (*Ovibos moschatus*) and sheep, demonstrating that this species can circulate among both wild and domestic ruminants inhabiting geographically isolated Arctic ecosystems. Notably, the phylogenetic analysis in that study revealed close genetic relationships among isolates from different host species, with no strict host-associated segregation, suggesting that *E. bovis* represents a generalist intestinal protist capable of colonizing multiple ruminant hosts across distinct ecological contexts.

Likewise, Huaman et al. (2022) reported the presence of *Entamoeba* spp., including *E. bovis* and related ribosomal lineages, in wild deer populations in Australia and demonstrated phylogenetic proximity between isolates obtained from wild cervids and domestic cattle. Their findings highlighted the potential for shared parasite populations between wildlife and livestock, even in ecosystems where direct interaction between host species may be limited. The phylogenetic clustering of wild and domestic isolates observed in that study closely mirrors the pattern identified in the present work, where *E. bovis* sequences from the Italian red deer clustered within the same lineage as those detected in cattle, sheep and goats, despite differences in host ecology and management systems.

In addition to the molecular characterization of isolates, the species-specific primers developed in this study targeting the 18S rRNA gene proved to be a reliable tool for the rapid identification of *E. bovis*. These primers consistently amplified a single, well-defined fragment of the expected size in all samples previously confirmed as *E. bovis* by sequencing, with no evidence of nonspecific amplification. The specificity of the assay was further confirmed by sequence analysis of the resulting amplicons, which showed complete or near-complete identity with reference *E. bovis* sequences available in GenBank. The development of species-specific primers represents a useful methodological advancement, as it enables rapid and accurate identification of *E. bovis* without the need for sequencing, which may be impractical in large-scale epidemiological studies or routine diagnostic settings. This approach is particularly valuable in cases where morphological identification alone may be inconclusive, such as in the presence of uninucleate cysts that may resemble immature stages of other *Entamoeba* species or morphologically similar amoebae. Therefore, the assay developed in this study may provide a practical tool for future surveillance, ecological and diagnostic investigations targeting *E. bovis* in domestic and wild ruminants.

Although direct transmission pathways cannot be inferred from the present data, the observed phylogenetic pattern is consistent with the existence of a broadly distributed and ecologically flexible *E. bovis* lineage capable of colonizing multiple ruminant hosts across diverse environmental settings. This may reflect either historical host sharing, ongoing environmental transmission or the circulation of genetically homogeneous populations maintained through indirect environmental exposure.

Despite providing the first molecular characterization of *E. bovis* in Italy, this study has some limitations that should be acknowledged. The sample size, particularly for certain species and locations, is relatively small and may not fully reflect the national prevalence or the complete genetic landscape of the parasite. As such, these findings should be considered preliminary, serving as a baseline for future, larger-scale epidemiological surveys aimed at mapping the distribution of *Entamoeba* species across broader geographical areas in both domestic and wild ruminant populations.

The integration of traditional microscopy with robust molecular techniques has become essential for resolving species boundaries, identifying cryptic taxa, and understanding the ecology and evolution of *Entamoeba* in ruminant systems. In the present study, the combined morphological and molecular characterization confirmed the occurrence of *E. bovis* in both domestic and wild ruminants in Italy, representing the first molecular evidence of this species in Italian livestock and in the endemic Italian red deer (*Cervus elaphus italicus*). Phylogenetic analysis demonstrated that isolates from domestic and wild hosts clustered within the same lineage, with no evidence of host-specific segregation, supporting the ecological flexibility and broad host range of this species.

## Data Availability

All the sequences generated in the present study are available in GenBank under the accession numbers: PZ097298-PZ097306.

## Figure 2 Long description

The image depicts a maximum-likelihood phylogenetic tree constructed from partial 18S rRNA gene sequences of Entamoeba species. The tree is oriented vertically, with branches extending from a common ancestor at the bottom to various species at the top. Bootstrap values greater than 50 percent are shown at the nodes, indicating the confidence level of the branching. Sequences obtained in this study are highlighted in a blue box and include BOV01FarmA, BOV02FarmB, SHE01FarmA, SHE02FarmB, CAP01FarmA, CAP02FarmB, CER01T1 and CER02T2. These sequences cluster within the Entamoeba bovis clade, alongside reference sequences from GenBank, such as KY012748.1 from a goat in Australia and FN666250.1 from a sheep in Sweden. Other notable sequences include Entamoeba sp. from various hosts and locations, such as Muscox from Greenland, human from Iraq and pig from Indonesia. The tree also includes sequences like Entamoeba chattoni from Japan and Entamoeba coli from the USA. The scale bar at the bottom represents genetic distance.

Navigate back to Figure 2.

## Table 1 Long description

The table lists eight successfully sequenced 18S rRNA samples and reports host species, sampling location, the closest BLAST match, percent identity, and reference and study accession numbers. Every sample’s best BLAST match is Entamoeba bovis. Sequence identity is consistently high, spanning 98.6 to 99.8 percent across all hosts and sites. Cattle samples from Farm A (urban) and Farm B (rural) show 99.4 and 99.0 percent identity, respectively. Sheep show the lowest identity in the dataset at 98.6 percent from Farm B, while the Farm A sheep sample is 99.3 percent. Goats include the highest identity observed, 99.8 percent at Farm A, and 98.9 percent at Farm B. Red deer from two reserves both show 99.8 percent identity. Accession numbers are provided for both the closest reference sequences and the sequences generated in this study, supporting traceability of each isolate.

Navigate back to Table 1.
